# Artificial intelligence in three-dimensional total-body photography for skin cancer surveillance

**DOI:** 10.3389/fmed.2026.1882075

**Published:** 2026-07-06

**Authors:** Kai Liang Lew, Kok Swee Sim, Wai Ti Chan

**Affiliations:** Faculty of Engineering and Technology, Multimedia University, Melaka, Malaysia

**Keywords:** artificial intelligence in dermatology, clinical readiness, external validation, melanoma, skin cancer surveillance, skin tone reporting, three-dimensional total body photography

## Abstract

Artificial intelligence is often used in studies that detect skin cancer, but most work uses images of selected lesions. These images are useful for classification. However, they do not fully match real screening situations. In lesion surveillance, clinicians need to inspect many lesions across the whole body and monitor whether lesions change over time. Three-dimensional total-body photography is able to capture a wider skin surface and can support lesion selection, lesion triage, risk assessment, and follow-up comparison. This approach gives artificial intelligence more opportunity to analyze the patient beyond one selected lesion. This Mini Review discusses recent evidence on artificial intelligence-assisted three-dimensional total-body photography for skin cancer surveillance. In current applications, this approach is also used in automated triage and lesion detection, multimodal risk prediction, phenotype extraction, and longitudinal tracking. The reviewed papers show early progress, but the evidence remains limited. Skin tone reporting, workflow integration, dataset transparency, false-positive and false-negative harm, cost, and equity remain key adoption issues. The objective is to focus on the shift from selected-lesion classification to whole-body imaging. Clinical value should not be judged only by lesion-level accuracy, but also by whether artificial intelligence-assisted three-dimensional total-body photography can improve patient-level surveillance across the whole skin surface and over time. Stronger prospective, longitudinal, workflow, cost, and equity evidence is still needed before routine clinical adoption in practice.

## 1 Introduction

Skin cancer detection is important in clinical practice because suspicious lesions are not always easy to recognize without expertise. Skin lesions may be melanoma or benign. Melanoma is a dangerous malignant skin cancer and can be fatal when diagnosed late (1). With the rise of artificial intelligence (AI), many studies now use this technology. These studies usually focus on image-based lesion classification and decision support for melanoma detection.

Most AI detection studies use cropped lesions or dermoscopic images. This does not fully match the earlier screening workflow in finding lesions across the whole body. In real clinical screening, clinicians need to examine many lesions and compare them with the patient's overall skin pattern. After that, they need to decide which lesions need further review. Clinicians therefore need not only lesion classification, but also lesion selection, prioritization, and follow-up.

Three-dimensional total-body photography (3D TBP) has changed the way AI can work in lesion detection. It can capture the wider skin surface and record multiple lesions on the body in a more structured way. It can support whole-body lesion detection, suspicious-lesion triage, patient-level risk assessment, and longitudinal monitoring. Existing review work has discussed AI applications in TBP and argued that TBP offers AI more opportunity in analyzing patients instead of only a single lesion image (2). Another scoping review also described 3D TBP as a tool that can document the skin surface and support early skin cancer detection (3).

**Figure 1:**
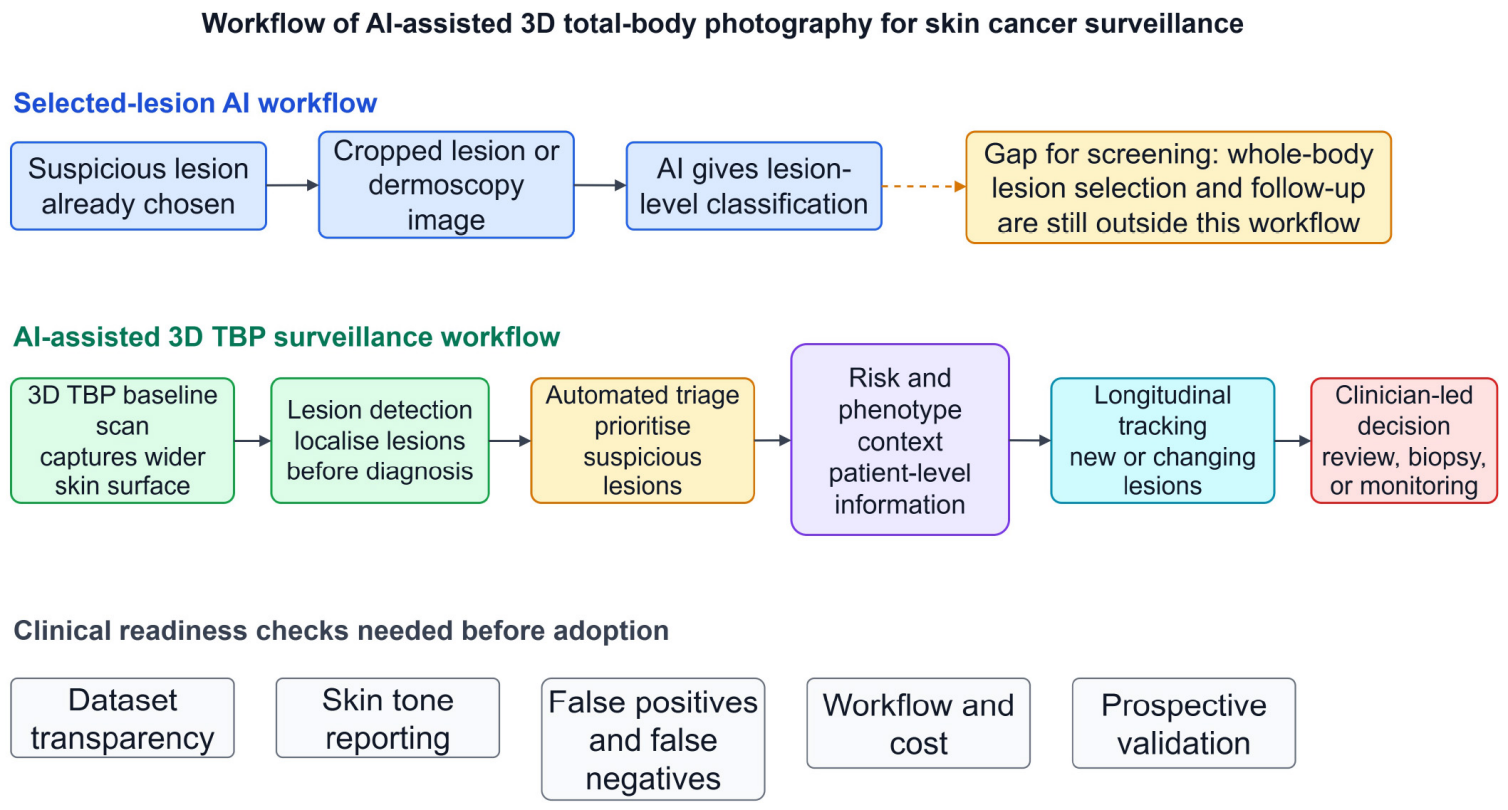
Workflow of AI-assisted three-dimensional total-body photography for skin cancer surveillance. The figure contrasts selected-lesion classification with whole-body capture, lesion detection, triage, phenotype extraction, risk prediction, longitudinal tracking, and clinician-led review.

The shift of AI focus from a single lesion to 3D TBP still needs focused discussion. Most research still focuses on classifying individual lesions. Less attention is given to how AI-assisted 3D TBP can help select lesions across the whole body. Patient-level risk assessment and longitudinal surveillance are also less discussed. This review combines recent evidence on AI-assisted 3D TBP. It focuses on automated triage, lesion detection, risk prediction, longitudinal lesion tracking, and clinical implementation challenges. Figure 1 shows the workflow of AI-assisted 3D TBP for skin cancer surveillance.

## 2 Literature search and selection

Papers were selected from 2024 to 2026. The search and local full-text check were last updated on 14 May 2026. Records were identified through targeted scholarly search and citation tracking, then checked against the final journal, publisher, DOI, and PubMed records where available. The search sources were Google Scholar, PubMed, Semantic Scholar, IEEE Xplore, and Crossref. Search strings combined terms for “skin cancer,” “melanoma,” “artificial intelligence,” “deep learning,” “three-dimensional total-body photography,” “total-body photography,” “3D TBP,” “lesion detection,” “lesion triage,” “risk prediction,” “lesion tracking,” “longitudinal monitoring,” “dataset transparency,” “skin tone,” “equity,” “workflow,” and “clinical validation.”

Inclusion criteria were paper publication years from 2024 to 2026. Papers had to be relevant to AI-assisted 3D TBP, total-body photography, non-dermoscopic 3D-TBP lesion images, longitudinal lesion monitoring, patient-level risk assessment, dataset transparency, workflow, or skin tone and equity issues in dermatology AI. Full text had to be available to classify the paper's role in this Mini Review. Exclusion criteria were papers published outside the years 2024 to 2026. Papers not relevant to the AI-assisted 3D TBP or skin cancer surveillance scope were excluded. Broad dermatology AI reviews that did not support the narrower 3D TBP focus were excluded. Papers without accessible full text and preprints were excluded. The lead author screened records while the co-authors settled ambiguous cases.

The initial search identified 1,804 records across five databases. It is based on a PRISMA-style selection flow as following:

- After de-duplication and removal of irrelevant records, 643 records remained.
- Title and abstract screening reduced the set to 235 papers, excluding records not relevant to AI-assisted 3D TBP or skin cancer surveillance.
- Further screening for full-text availability and direct relevance reduced the set to 30 full-text papers, excluding broad reviews, lack of 3D context, preprints, or publication outside the range.

Table 1 shows the section-level coverage of the 30 papers used in this Mini Review.

**Table 1:** Section-level coverage of the 30 papers used for this Mini Review.

Main section/theme	Primary papers	Direct 3D TBP	Main use in this review
Introduction and scope framing	4	2	Shows that skin cancer AI, AI skin imaging, and 3D TBP have been reviewed, but the selected-lesion to surveillance shift needs a narrower Mini Review.
From single-lesion AI to whole-body imaging	4	2	Explains why selected-lesion AI is not enough for real screening and why whole-body context matters.
Current AI applications in 3D TBP	6	6	Summarizes triage, lesion detection, multimodal risk prediction, phenotype extraction, and 3D-TBP-based classifiers.
Longitudinal monitoring and lesion tracking	4	4	Covers annotation, repeated imaging, lesion tracking, follow-up support, and longitudinal datasets.
Challenges for clinical use	10	6	Covers transparency, skin tone, false predictions, workflow integration, remote assessment, and equity.
Discussion and future directions	2	1	Positions foundation models and deep imaging phenotypes as future directions.
Total	**30**	**21**	**Current evidence pool used to support the Mini Review argument**.

Each paper is assigned to one main section to avoid double counting in the evidence map.

## 3 From single-lesion to whole-body imaging

A single-lesion image is usually just a cropped image of one lesion. The image is useful for AI to learn and analyze the visual pattern of the lesion. The weakness is that it only works after a lesion has already been selected. For real screening, that is not practical. The clinician still needs to find the suspicious lesion across the patient's whole body, which can be time-consuming. AI also cannot assess the full body if it is only trained on selected-lesion images. 3D TBP can capture the full body of the patient, and AI can be used to help identify and prioritize suspicious lesions for further analysis (4). It may reduce the clinician's time for manual inspection and help focus attention on lesions that need closer review. The clinician also compares lesions on the same patient. If a lesion looks different from the patient's other lesions, it may need closer attention. Clinically, this comparison is known as the ugly duckling concept (5). 3D TBP images are limited to ≈0.1 mm/pixel resolution. Dermoscopy offers much higher resolution, < 0.01 mm/pixel. 3D TBP is effective for wide-field triage and mapping. However, it cannot replace dermoscopy for viewing microscopic structures like atypical pigment networks.

The SLICE-3D dataset shows how lesion crops can be extracted from 3D TBP and used for skin cancer detection (6). It connects whole-body imaging with lesion-level AI analysis. Multimodal datasets can further combine lesion images with clinical and patient-level information (7). This widens the direction of AI from classifying one image to using body-level and clinical patient-level information to improve the screening workflow.

The shift from AI analyzing single lesions to using 3D TBP is more than a new image type. It is a change in the screening workflow. It can assist in finding and ranking lesions on a patient's body and watching them over time.

## 4 Current AI applications in 3D TBP

The main applications of AI in 3D TBP are automated triage, lesion detection, risk prediction, and phenotype extraction. The AI applications move beyond single-lesion classification to support whole-body surveillance across visits. These applications can help clinicians review 3D TBP images more efficiently. Automated triage can prioritize suspicious lesions that may need closer clinical review (8). Lesion detection is also important because the suspicious lesion needs to be found before diagnosis can happen. This may reduce the burden of the traditional screening workflow, which depends on clinicians manually checking many lesions across the patient body (9).

Multimodal AI means the model uses more than one type of information, such as 3D images, lesion features, and clinical patient data. Skin cancer assessment does not depend on image appearance only. Clinical information and patient-level features can also support risk prediction (10).

AI using 3D TBP can analyze broader skin features beyond individual lesions. This includes classifying site-specific photodamage (11) and identifying patient-level phenotypes that may indicate melanoma risk (12). AI in 3D TBP is focusing more into scanning broader skin patterns and risk assessment rather than classifying selected-lesion images. Rashid et al. (13) proposed a ResNet-18 based model to classify skin cancer with 3D TBP images. Their results showed that their model outperforms the other models. However, it is not guaranteed that a strong performance model can be ready for clinical workflow.

AI has started to advance the 3D TBP area to make it capable of performing more complex tasks, including triage, detection, multimodal risk prediction, and phenotype analysis. While progress is advancing, the evidence is not consistent. Triage and lesion detection are clearly linked to 3D TBP. However, phenotype extraction and risk prediction are not yet proven clinical tools. These show that they are still in a preliminary stage.

Current AI methods for 3D TBP use convolutional neural networks (CNNs) and achieve an area under the curve (AUC) of 0.94–0.96 for classifying lesions (1). CNNs extract local lesion features. ViTs use self-attention to understand global body context, which is crucial for identifying ‘ugly duckling' outliers. Vision transformers (ViTs) have improved this performance to 0.96–0.98. The ISIC 2024 challenge used over 900,000 lesion images from 3D TBP. SLICE-3D has 2D lesion images, not 3D data. ISIC 2024 provides extensive patient IDs, perfect for validating patient-level triage algorithms. The models that won the challenge used the “ugly duckling” concept (8). Foundation models, like PanDerm, were trained on over two million skin images. It outperformed clinicians by 10.2% in detecting early melanoma (14). These advancements show a clear shift from AI that classifies single lesions to AI that evaluates the whole body of patients.

## 5 Longitudinal monitoring and lesion tracking

Longitudinal monitoring is important because skin cancer surveillance is not a one-time task, and it needs to continue monitoring the skin cancer for a period. Clinicians need to know whether the lesion is new, changing, or stable. Therefore, a single image cannot prove this, as it only captures the lesion at a specific time.

3D TBP allows a structured scan comparison. This is because after an initial full-body scan, the next scan can be compared with the previous scan (15). This allows the clinicians to assess the lesions of the patients across their visits. It is very useful for longitudinal monitoring (16).

For AI, this also changes the dataset requirements. A model trained only on one image of a lesion can mainly learn the appearance. To learn change, AI needs repeated images, lesion labels, and clinical information across visits. Annotation is important because it tells the model which lesion or image region should be learned and compared (17). Longitudinal datasets can then support research on how lesions change across visits and how these changes relate to skin cancer detection (18). Tracking lesions over visits is challenging for computer vision. Issues include 3D registration errors from posture changes and complex lesion matching. Registration coordinates and visual descriptors must be optimized for correct correspondence, especially with many lesions.

This is why longitudinal monitoring is one of the strongest arguments for AI-assisted 3D TBP. It moves the task from one-time lesion classification to follow-up support, lesion tracking, and change-based surveillance. At present, this is a stronger conceptual and dataset-driven argument than a proven clinical outcome claim, and more studies are needed to show whether AI-assisted lesion tracking can improve real clinical outcomes.

## 6 Challenges for clinical use

Dataset transparency is one of the main challenges for AI in 3D TBP. The data source, patient background, lesion labels, and data limitations should be clearly reported. AI performance depends strongly on whether the dataset is representative and of good quality. This is also related to ethical data use. Patient images and clinical information should be collected, stored, and shared properly. If skin tone, patient background, or lesion labels are not clearly reported, clinicians and researchers cannot judge if the dataset is complete. They also cannot judge if it is suitable for training and evaluating artificial intelligence models (19).

Skin tone is also important in the dataset. This is because it can affect how lesions appear in images. If the dataset does not include enough diversity, AI may not perform equally well for all patient groups. This can affect the fairness and generalizability of dermatology AI (20). While automated skin tone analysis is promising, it requires rigorous validation before clinical use (21). Therefore, skin tone reporting should not be treated as a small detail. It is essential for developing safe and equitable AI.

False positives and false negatives are major AI challenges. AI can produce wrong predictions because of model limitations, dataset imbalance, poor image quality, or weak annotation. More detailed annotations can provide better information for AI to learn from. If AI produces too many false positives, clinicians may receive too many unnecessary alerts, which can lead to further review, biopsy, or excision of benign lesions. False negatives are more dangerous because the model may miss a suspicious lesion, delay diagnosis, and worsen patient outcomes. Many false alarms cause severe cognitive overload and alert fatigue. This may lead clinicians to miss critical lesions. Therefore, AI image analysis should not be used as a stand-alone assessment of skin tumors (22).

AI-assisted 3D TBP needs seamless clinical integration for proper use. It must fit into existing clinical workflows for image handling and decision-making.

In a practical dermatology workflow, trained staff could first capture the 3D images, and AI could then pre-mark suspicious lesions, support lesion detection, or help identify changing lesions before clinician review (8, 9, 16). The clinician would still decide whether a lesion needs dermoscopy, biopsy, short-term follow-up, or routine monitoring. These systems are Software as a Medical Device (SaMD), creating regulatory hurdles. Locked algorithms are static after deployment so the adaptive models need specialized Pre-Determined Change Control Plans (PCCPs). The AI output also needs to be stored with the clinical record and compared with the next TBP session (23).

If this process is not clear, AI may become difficult to use even when the model itself performs well (23). Remote evaluation using 3D TBP can support dermatology assessment. However, image-based review still has limitations. It may not fully replace in-person clinical examination (24). Studies comparing 3D TBP with traditional clinical and dermoscopic examination show that it should be understood as a support tool. It is not a replacement for standard clinical assessment (25).

Clinician trust is crucial. If an AI system outputted too many false predictions, clinicians could not trust the system. A clinician who is overconfident in AI performance is very dangerous. This is because if it gives false negative results on a patient's lesion, it could be fatal. Therefore, AI for 3D TBP should assist the clinicians and not replace clinical judgment. There is no clear law for diagnostic error responsibility. When AI systems perform like or better than skin doctors, who is responsible for errors gets confusing. Doctors, hospitals, and AI companies might all be responsible. Clear legal rules for this are still missing. Hobelsberger et al. (26) shows that 3D-TBP assessment has difficulty in interpreting the lesion when it is reviewed from the image sectors but not from the person. Prospective validation, cost, and equity are still in major gaps. 3D TBP systems like Vectra WB360 are expensive. This limits the usage only in rich city hospitals, which may be unfair for others to access. AI-assisted 3D TBP is only cost-effective for high-risk patients, like those with atypical mole syndrome. It is not viable for general screening.

Multiple AI studies demonstrated good performance in the dataset, but could not improve the real workflow. There are several limitations in 3D TBP, such as the images have lower resolution than dermoscopy, which can limit the lesion classification performance (17). Some body areas are not fully visible in 3D TBP (15). In the ISIC 2024 dataset, only 0.1% of lesions were malignant (8). Tjiu and Lu (27) shows that AI achieved an AUC of 0.89 on lighter skin tone while it achieved 0.82 on darker skin tones.

**Figure 2:**
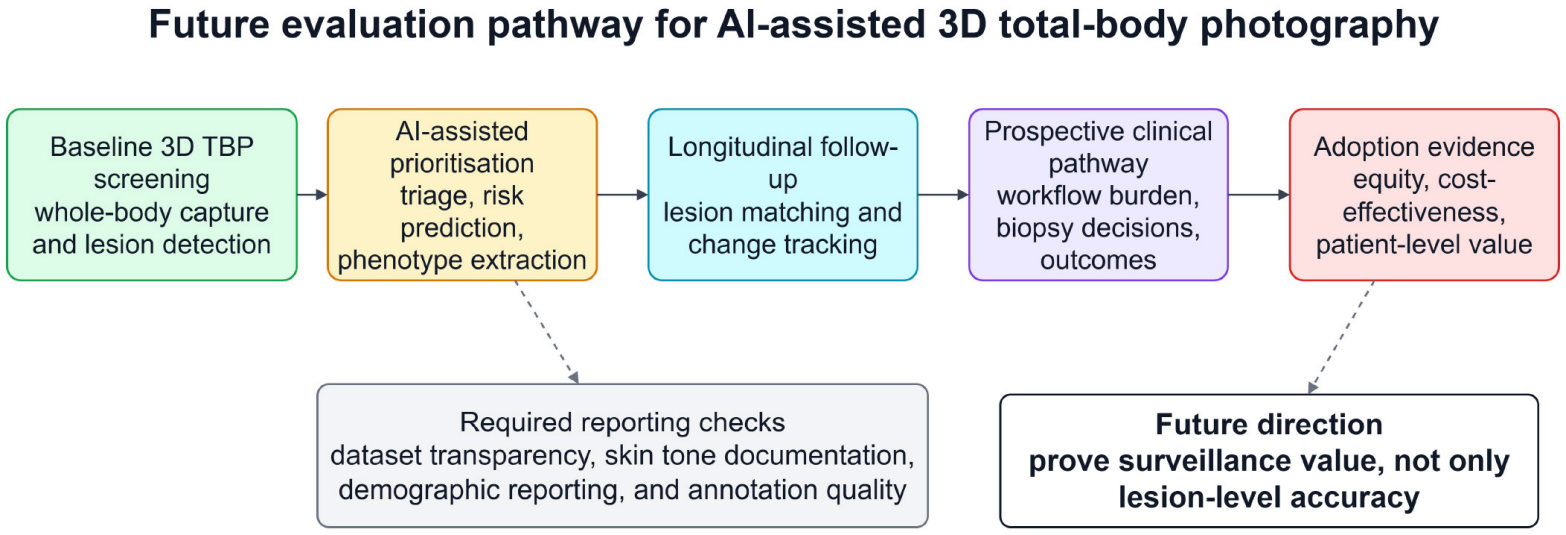
Clinical readiness checks for AI-assisted 3D TBP. The figure summarizes the main evidence areas that remain weak before clinical adoption, including dataset transparency, skin tone and equity, prediction harm, workflow integration, cost and access, and prospective validation.

The implementation of 3D TBP for practical usage will face several challenges. Current AI systems must use graphics processing unit (GPU) to process a patient in 70 seconds while central processing unit (CPU) takes up to 6 min (8). AI flagged 93% of healthy patients with incorrect lesions in clinical tests. This may give burden to the clinicians (22). Without these changes, AI-assisted 3D TBP might stay in research settings. Figure 2 shows the main evidence areas that remain weak before clinical adoption.

## 7 Discussion and future directions

AI 3D TBP is more than just another image classification task. Traditional skin cancer AI usually focuses on whether a model can classify one selected lesion correctly. 3D TBP changes this focus. The more important issue is whether AI can support patient-level surveillance by helping clinicians detect, prioritize, and monitor lesions across the whole skin surface over time.

Across the reviewed papers, AI is strong when using it to classify images. Automated triage with 3D TBP can rank suspicious lesions. However, its clinical utility, optimal thresholds, cost, and risk of overdiagnosis require further evaluation. It is still weak when it comes to patient outcomes, cost-effectiveness, or independent clinical decision-making (8). Multimodal risk prediction is promising but needs validation using real-world TBP data and independent external patient groups (10). Lesion tracking aids follow-up, but its impact on patient outcomes remains to be proven (16, 18). Table 2 shows the evidence matrix for all 30 papers.

**Table 2:** Evidence matrix for the 30 papers used in this Mini Review.

Study	Evidence / reviewed focus	Review role	Main limitation for clinical readiness
Górecki et al. (1)	Reviews artificial intelligence and new technologies in melanoma diagnosis.	Introduction / background	Broad melanoma AI review; not specific to AI-assisted 3D TBP surveillance.
Wei et al. (29)	Reviews artificial intelligence and skin cancer.	Introduction/ background	Broad skin cancer AI review; not specific to AI-assisted 3D TBP surveillance.
Primiero et al. (2)	Reviews opportunities and challenges for AI skin image analysis using total body photography.	Scope framing	Broad TBP-AI review; does not focus only on the current surveillance workflow.
Baete et al. (3)	Scoping review of 3D TBP for early skin cancer detection.	Scope framing	Reviews 3D TBP as a clinical imaging innovation, but AI-specific evidence is still limited.
Kurtansky et al. (6)	Describes the SLICE-3D dataset with lesion crops extracted from 3D TBP for skin cancer detection.	Whole-body to lesion-level AI	Dataset supports lesion-level modeling, but clinical pathway benefit still needs validation.
Useini et al. (5)	Develops self-supervised screening around suspicious lesions and ugly-duckling-style patient comparison.	Whole-body screening logic	Useful for patient-level comparison, but not a direct 3D TBP clinical deployment study.
Ferreirinha et al. (4)	Discusses melanoma diagnosis with 3D total-body photography.	3D TBP modality background	Supports the imaging modality, but not a primary AI validation study.
Chiou et al. (7)	Presents a multimodal image dataset for AI-based skin cancer benchmarking.	Multimodal dataset context	Strong for multimodal AI direction, but not specific to 3D TBP surveillance.
Kurtansky et al. (8)	Studies automated triage of cancer-suspicious skin lesions with 3D total-body photography.	AI triage	Strong core AI application, but clinical workflow and prospective outcome evidence remain early.
Saha et al. (9)	Provides skin region images extracted from 3D total body photographs for lesion detection.	Lesion detection/localization	Dataset and detection resource; not proof that deployment improves patient outcomes.
Wang et al. (10)	Uses explainable multimodal AI with 3D imaging and clinical data for lesion risk prediction.	Multimodal risk prediction	Promising multimodal method, but still needs broader external and prospective validation.
Kahler et al. (11)	Classifies site-specific cutaneous photodamage using CNNs and 3D TBP.	Phenotype extraction	Shows broader skin feature analysis, but not direct skin cancer triage outcome evidence.
Kahler et al. (12)	Identifies 3D-TBP-derived cutaneous phenotypes associated with late-onset invasive melanoma risk.	Patient-level risk phenotype	Supports risk stratification, but translation into AI-guided screening pathways remains open.
Rashid et al. (13)	Tests a ResNet-18 approach for skin cancer classification with 3D TBP images.	3D-TBP classifier example	Dataset performance should not be treated as clinical readiness.
Primiero et al. (17)	Proposes an annotation protocol for total body photography in machine learning.	Annotation / tracking support	Protocol paper; it supports dataset quality needs but does not validate clinical outcomes.
Huang et al. (16)	Revisits lesion tracking in 3D total body photography.	Longitudinal lesion tracking	Core tracking paper, but real-world outcome benefit still needs clinical testing.
Hobelsberger et al. (15)	Studies 3D TBP, digital dermoscopy, and reflectance confocal microscopy for high-risk melanoma follow-up.	Clinical follow-up support	Clinical follow-up evidence, but not an AI-only study.
Ghahari et al. (18)	Provides a longitudinal dataset of tile and dermoscopic images with metadata for identifying skin cancers.	Longitudinal dataset	Enables change-based research, but algorithmic and clinical validation remain separate tasks.
Li et al. (19)	Applies a dataset nutrition label for dermatologic AI transparency.	Dataset transparency	Comment/resource focus; does not directly evaluate AI-assisted 3D TBP performance.
Sitaru et al. (24)	Studies remote evaluation of general skin diseases using 3D TBP.	Remote workflow	Supports remote assessment context, but image-based review cannot fully replace clinical examination.
Parvathaneni et al. (23)	Surveys dermoscopic image capture, storage, and AI integration practices.	Image management/workflow	Workflow-relevant but only indirectly related to 3D TBP skin cancer surveillance.
Gellrich et al. (25)	Compares skin examination using 3D TBP with clinical and dermoscopic examination.	Clinical comparison	Supports 3D TBP limits and value, but not AI validation.
Weir et al. (20)	Evaluates skin tone scales for dermatologic dataset labeling.	Skin tone reporting	Important for equity, but performance across clinical 3D TBP pathways remains to be shown.
Ulrich et al. (21)	Studies automated AI-based skin tone analysis in dermatological patients.	Skin tone automation	Supports documentation, but needs cautious validation before routine use.
Weir et al. (30)	Surveys skin tone assessment in prospective research.	Skin tone methodology	General skin tone evidence; not specific to 3D TBP skin cancer AI.
Hobelsberger et al. (26)	Tests clinician ability to identify non-melanoma skin cancer on 3D-TBP sectors.	Clinical interpretation limits	Shows image-sector assessment difficulty; does not validate autonomous AI use.
Tjiu and Lu (27)	Reviews equity and generalizability of AI for skin-lesion diagnosis.	Equity/generalizability	Broad skin-lesion AI evidence; not specific to 3D TBP.
Lallas et al. (22)	Argues AI-based image analysis is insufficient as a stand-alone assessment in real clinical practice.	AI safety / caution	Strong caution evidence, but not 3D-TBP-specific.
Yan et al. (14)	Presents a multimodal vision foundation model for clinical dermatology.	Future multimodal AI	Future direction evidence; not directly a 3D TBP surveillance validation study.
Kahler et al. (28)	Discusses the deep imaging phenotype for melanoma risk stratification.	Future risk stratification	Conceptual risk-stratification direction; clinical implementation still needs testing.
Górecki et al. (1)	Reviews artificial intelligence and new technologies in melanoma diagnosis.	Introduction / background	Broad melanoma AI review; not specific to AI-assisted 3D TBP surveillance.
Wei et al. (29)	Reviews artificial intelligence and skin cancer.	Introduction/background	Broad skin cancer AI review; not specific to AI-assisted 3D TBP surveillance.
Primiero et al. (2)	Reviews opportunities and challenges for AI skin image analysis using total body photography.	Scope framing	Broad TBP-AI review; does not focus only on the current surveillance workflow.
Baete et al. (3)	Scoping review of 3D TBP for early skin cancer detection.	Scope framing	Reviews 3D TBP as a clinical imaging innovation, but AI-specific evidence is still limited.
Kurtansky et al. (6)	Describes the SLICE-3D dataset with lesion crops extracted from 3D TBP for skin cancer detection.	Whole-body to lesion-level AI	Dataset supports lesion-level modeling, but clinical pathway benefit still needs validation.
Useini et al. (5)	Develops self-supervised screening around suspicious lesions and ugly-duckling-style patient comparison.	Whole-body screening logic	Useful for patient-level comparison, but not a direct 3D TBP clinical deployment study.
Ferreirinha et al. (4)	Discusses melanoma diagnosis with 3D total-body photography.	3D TBP modality background	Supports the imaging modality, but not a primary AI validation study.
Chiou et al. (7)	Presents a multimodal image dataset for AI-based skin cancer benchmarking.	Multimodal dataset context	Strong for multimodal AI direction, but not specific to 3D TBP surveillance.
Kurtansky et al. (8)	Studies automated triage of cancer-suspicious skin lesions with 3D total-body photography.	AI triage	Strong core AI application, but clinical workflow and prospective outcome evidence remain early.
Saha et al. (9)	Provides skin region images extracted from 3D total body photographs for lesion detection.	Lesion detection/localization	Dataset and detection resource; not proof that deployment improves patient outcomes.
Wang et al. (10)	Uses explainable multimodal AI with 3D imaging and clinical data for lesion risk prediction.	Multimodal risk prediction	Promising multimodal method, but still needs broader external and prospective validation.
Kahler et al. (11)	Classifies site-specific cutaneous photodamage using CNNs and 3D TBP.	Phenotype extraction	Shows broader skin feature analysis, but not direct skin cancer triage outcome evidence.
Kahler et al. (12)	Identifies 3D-TBP-derived cutaneous phenotypes associated with late-onset invasive melanoma risk.	Patient-level risk phenotype	Supports risk stratification, but translation into AI-guided screening pathways remains open.
Rashid et al. (13)	Tests a ResNet-18 approach for skin cancer classification with 3D TBP images.	3D-TBP classifier example	Dataset performance should not be treated as clinical readiness.
Primiero et al. (17)	Proposes an annotation protocol for total body photography in machine learning.	Annotation/tracking support	Protocol paper; it supports dataset quality needs but does not validate clinical outcomes.
Huang et al. (16)	Revisits lesion tracking in 3D total body photography.	Longitudinal lesion tracking	Core tracking paper, but real-world outcome benefit still needs clinical testing.
Hobelsberger et al. (15)	Studies 3D TBP, digital dermoscopy, and reflectance confocal microscopy for high-risk melanoma follow-up.	Clinical follow-up support	Clinical follow-up evidence, but not an AI-only study.
Ghahari et al. (18)	Provides a longitudinal dataset of tile and dermoscopic images with metadata for identifying skin cancers.	Longitudinal dataset	Enables change-based research, but algorithmic and clinical validation remain separate tasks.
Li et al. (19)	Applies a dataset nutrition label for dermatologic AI transparency.	Dataset transparency	Comment/resource focus; does not directly evaluate AI-assisted 3D TBP performance.
Sitaru et al. (24)	Studies remote evaluation of general skin diseases using 3D TBP.	Remote workflow	Supports remote assessment context, but image-based review cannot fully replace clinical examination.
Parvathaneni et al. (23)	Surveys dermoscopic image capture, storage, and AI integration practices.	Image management/workflow	Workflow-relevant but only indirectly related to 3D TBP skin cancer surveillance.
Gellrich et al. (25)	Compares skin examination using 3D TBP with clinical and dermoscopic examination.	Clinical comparison	Supports 3D TBP limits and value, but not AI validation.
Weir et al. (20)	Evaluates skin tone scales for dermatologic dataset labeling.	Skin tone reporting	Important for equity, but performance across clinical 3D TBP pathways remains to be shown.
Ulrich et al. (21)	Studies automated AI-based skin tone analysis in dermatological patients.	Skin tone automation	Supports documentation, but needs cautious validation before routine use.
Weir et al. (30)	Surveys skin tone assessment in prospective research.	Skin tone methodology	General skin tone evidence; not specific to 3D TBP skin cancer AI.
Hobelsberger et al. (26)	Tests clinician ability to identify non-melanoma skin cancer on 3D-TBP sectors.	Clinical interpretation limits	Shows image-sector assessment difficulty; does not validate autonomous AI use.
Tjiu and Lu (27)	Reviews equity and generalizability of AI for skin-lesion diagnosis.	Equity/generalizability	Broad skin-lesion AI evidence; not specific to 3D TBP.
Lallas et al. (22)	Argues AI-based image analysis is insufficient as a stand-alone assessment in real clinical practice.	AI safety / caution	Strong caution evidence, but not 3D-TBP-specific.
Yan et al. (14)	Presents a multimodal vision foundation model for clinical dermatology.	Future multimodal AI	Future direction evidence; not directly a 3D TBP surveillance validation study.
Kahler et al. (28)	Discusses the deep imaging phenotype for melanoma risk stratification.	Future risk stratification	Conceptual risk-stratification direction; clinical implementation still needs testing.

Based on current research, most of studies use existing datasets, or proof-of-concept tests. There are only a few studies that validate findings with actual patients and no studies have proven improved patient results in routine screening.

Future studies need to go beyond just dataset performance. High accuracy on a dataset is useful, but it is not enough to show clinical value. 3D TBP is valuable for repeated whole-body assessment, not just single lesion detection. It needs to test AI-assisted 3D TBP in actual screening pathways, including its effect on workload, unnecessary procedures, missed lesions, patient access, and cost. Equity is also critical. This is because AI that works well only for certain skin tones, datasets, or clinical settings could worsen healthcare gaps (27). Therefore, AI-assisted 3D TBP should be assessed not only by accuracy, but also by transparency, safety, workflow integration, cost, and equity.

Another future direction is the use of multimodal and foundation models in dermatology. These models can help combine different types of information to provide a more comprehensive assessment. However, these models still require validation on clinical workflow (14). Deep imaging phenotypes can link visible skin patterns to melanoma risk (28). AI should support prioritization, risk assessment, and long-term monitoring. Future research should also make models more understandable using explainable AI. This helps doctors trust and understand AI decisions. Future research also needs strong multicenter studies. This will test if the findings work in different clinics, with various hardware, and for diverse patients. These studies are important before AI-assisted 3D TBP is used in daily practice.

## 8 Conclusion

AI in 3D TBP shifts the skin cancer area from selected-lesion classification to whole-body surveillance. It helps clinicians in detecting, prioritizing, and monitoring all lesions over time. This can help clinicians reduce their burden. Current studies support early applications in automated triage, lesion detection, multimodal risk prediction, phenotype extraction, and longitudinal lesion tracking.

The clinical value will depend on whether future studies can show benefit in real screening pathways. Overall, 3D TBP provides more opportunities for AI to explore and develop a more patient-level and surveillance-focused application. However, these AI applications need to prove that they are effective in clinical workflow before being adopted into routine skin cancer care.
